# Significance of nanoparticle’s radius, heat flux due to concentration gradient, and mass flux due to temperature gradient: The case of Water conveying copper nanoparticles

**DOI:** 10.1038/s41598-021-81417-y

**Published:** 2021-01-21

**Authors:** Nehad Ali Shah, I. L. Animasaun, Jae Dong Chung, Abderrahim Wakif, F. I. Alao, C. S. K. Raju

**Affiliations:** 1 Informetrics Research Group, Ton Duc Thang University, Ho Chi Minh City, Vietnam; 2 Faculty of Mathematics and Statistics, Ton Duc Thang University, Ho Chi Minh City, Vietnam; 3 Department of Mathematical Sciences, Fluid Dynamics and Survey Research Group, Federal University of Technology, Akure, PMB 704 Nigeria; 4 Department of Mechanical Engineering, Sejong University, Seoul, 05006 South Korea; 5 Laboratory of Mechanics, Faculty of Sciences Ain Chock, University Hassan II of Casablanca, 20000 Casablanca, Morocco; 6 Department of Mathematics, GITAM Bengaluru, Bangalore, Karnataka 562163 India

**Keywords:** Mathematics and computing, Physics

## Abstract

The performance of copper selenide and effectiveness of chemical catalytic reactors are dependent on an inclined magnetic field, the nature of the chemical reaction, introduction of space heat source, changes in both distributions of temperature and concentration of nanofluids. This report presents the significance of increasing radius of nanoparticles, energy flux due to the concentration gradient, and mass flux due to the temperature gradient in the dynamics of the fluid subject to inclined magnetic strength is presented. The non-dimensionalization and parameterization of the dimensional governing equation were obtained by introducing suitable similarity variables. Thereafter, the numerical solutions were obtained through shooting techniques together with 4th order Runge–Kutta Scheme and MATLAB in-built bvp4c package. It was concluded that at all the levels of energy flux due to concentration gradient, reduction in the viscosity of water-based nanofluid due to a higher radius of copper nanoparticles causes an enhancement of the velocity. The emergence of both energy flux and mass flux due to gradients in concentration and temperature affect the distribution of temperature and concentration at the free stream.

## Introduction

The roles of energy and mass fluxes due to temperature and concentration gradients play major roles in electrochemical processes, dynamics of gases, chemical catalytic reactors, production of copper selenide sequel to the pioneering work by Louis Dufour^1^; see Ingle and Horne^2^, Hollinger and Lucke^3^, Korzhuev^4^, Kim et al.^5^, Mahdy^6^, Anjum Badruddin^7^. Conservation of energy is capable to unravel the nature of outward flux of energy. Meanwhile, Hort et al.^8^ once remarked that concentration currents and heat are two driven forces of fluctuations in temperature as in the case of conservation of energy. The effect of energy flux due to concentration gradient on six different gases ($CO_2+O_2$, $N_2+H_2$, $H_2+CO_2$, $N_2+O_2$, $CO_2+N_2$, $H_2+O_2$) at various levels of pressure was examined experimentally by Rastogi and Madan^9^. It was remarked that kinetic theory is useful to determine the Dufour coefficient of each gas and temperature difference within the domain $(T_w - T_\infty )$ is dependent on pressure that maintains the motion of each gas. Formation of energy flux due to temperature gradient is highly significant in gases than in liquids. In fact, the occurrence in a less viscous fluid is 10,000 larger than in high viscous fluid. This fact suggests that the magnitude of the Dufour number is larger for gases (Hort et al.^10^). Dynamics of peristaltic flow through a channel of width 2*a* subject to energy flux due to concentration gradient and mass flux due to energy gradient was closely examined by Hayat et al.^11^. It was shown that temperature distribution is an increasing property of both Dufour and Soret effects. Although, the increase in the temperature distribution is more enhanced near the surface. The reverse is the observed effects of Dufour and Soret phenomena on the concentration as both properties decreases.

Further examination of energy and mass fluxes by Linz^12^ led to a conclusion that a larger Dufour number was recommended for gaseous mixtures in which energy flux due to concentration gradient is significant. Following the suggestion by Partha et al.^15^, Lewis number is more appropriate than Schmidt number in characterizing heat and mass transfer due to the significance of energy flux due to concentration gradient and mass flux due to temperature gradient. This led to a robust analysis of the interconnectedness of Lewis number, Dufour number, and Soret number. The results show that when $L_e = 0.1$ and $S_r = 0.6$, Nusselt number $Nu_xRa_x^{-0.5}$ decreases with Dufour effect at the rate of $-0.076145399$. But, when Lewis number is more higher (i.e. $L_e = 1$) and Soret number still remain $S_r = 0.6$, the same Nusselt number $Nu_xRa_x^{-0.5}$ decreases with Dufour effect at higher rate of $-0.585851817$. Energy flux due to concentration gradient was pointed out by Garcia-Colin et al.^13^ as the most important source of heat conduction within the region where magnetic fields do not exist or even weak. The further associated increase in effective thermal conductivity to growth in Dufour effect. The major reason why temperature increases due to higher magnetic strength were associated with the fact that heat conduction in the perpendicular direction diminishes whenever magnetic field is intensified. The conclusions mentioned above are not affected in the correction published as Ref.^14^.

Enhancement in the transfer of heat across fluid flow has made experts in thermal engineering embrace the efficiency of nanofluids. The earlier mentioned advancement is based on the nature of the base fluid and nanoparticles. Nanoparticle concentration and temperature effects on the ratio of mass to density and viscosity are some of the physical properties. However, thermal conductivity and specific heat capacity at different levels of concentration of nanoparticles, nanoparticles’ size, and temperature are some of the thermal properties. The concentration of nanoparticles, pressure drop, friction factor, nanoparticles radius are also some of the outlined characteristics of nanofluids as pointed out by Mohamoud Jama^16^, Narayanan and Rakesh^17^, Lin and Yang^18^. Intrinsic magnetic related properties of magnetite vary as to its diameter changes (i.e. higher ratio of surface to volume). As the size of nanoparticles becoming smaller, the superparamagnetic nature of magnetic nano-sized particles even changes^19^. Ashraf et al.^20^ remarked that changes in the radius of nanoparticles affect the characteristics of both interphase and nanoparticles. According to Yapici et al.^21^, the outcome of comparative analysis of ethylene glycol conveying SiO$_2$ (20–30 nm, 60–70 nm), TiO$_2$ (30 nm, 50 nm), ZnO (10–30 nm, 35–45 nm, 80–200 nm), CuO (40 nm, 80 nm), and MgO (20 nm, 40 nm) shows that the relative viscosity of these nanofluids is a decreasing property of particle sizes. Vishal et al.^22^ noted that the viscosity of nanofluid can be greatly influenced by particle size, the nature of energy transfer between fluid’s layer and the surface of the particles. There exist significant changes in the melting point of nanoparticles due to rises in the radius. This conclusion was based on the fact that the melting temperature was seen to be a decreasing property of higher particle size with a significant decreasing rate between spherical and nanoparticles; see Antoniammal and Arivuoli^23^.

The analysis of Namburu et al.^24^ indicates that increasing the diameter of silicon dioxide nanoparticles in ethylene glycol and water corresponds to a decrease in the nanofluid’s viscosity. It is worth remarking that the observed decrease in fluid’s viscosity is more significant when the nanofluid is cold (negative temperature). At a larger temperature, the decrease in viscosity with particle size disappears. In the case of a nanofluid parallel to a moving stretchable sheet, platelet shape of molybdenum disulfide (MoS2) nanoparticles was found by Hamid et al.^25^ to produce a unique heat transfer. In another study, Sheikholeslami et al.^26^ illustrated the movement of multiple wall carbon nanotube and Iron(iii)oxide nanoparticles in a typical water based nanofluid through a porous medium when Lorentz force is predominant. The dynamics of water conveying alumina and copper nanoparticles over a split lid-driven square cavity was examined by Khan et al.^27^ and it was shown that higher Nusselt number proportional to the heat transfer rate is attained at the point when both lids meet. In another related report by Khan et al.^28^, carbon nanotubes and heating of the wall are two factors capable to boost the local Nusselt number in the case of water-based carbon nanotubes over a right-angle trapezoidal cavity. Meanwhile, the local Nusselt numbers are increasing property of solid volume fraction of carbon nanoparticles; Hamid et al.^29^. The analysis of seven different hybrid nanofluids by Nehad et al.^30^ shows that optimal Nusselt number is achievable when suction and stretching are significantly large but less dense nanoparticles like silicon dioxide and multiple wall CNT.

Sequel to the aforementioned reviews of related literatures, in the presence of Joule heating and space-dependent heating, it is noteworthy to examine the significance of increasing nanoparticle radius and inclined magnetic field on the dynamics of chemical reactive water conveying copper nanoparticles through a porous medium. The outcome of such study would be very helpful to experts dealing with the performance of copper selenide and effectiveness of chemical catalytic reactors. This study was designed to provide answers to the following related research questions:

1. when energy flux due to concentration gradient and mass flux due to temperature gradient are negligible, what is the significance of increasing radius of nanoparticles on the local skin friction coefficients, heat transfer rate, and mass transfer rate?
2. At various levels of energy flux and mass flux due to gradients in concentration and temperature respectively, how does increasing radius of copper nanoparticles influences the transport phenomena of *Cu*-water nanofluids?
3. What is the variation in $Cf_x\sqrt{Re_x}$, $\frac{Nu_x}{\sqrt{Re_x}}$, and $\frac{Sh_x}{\sqrt{Re_x}}$, with increasing radius of nanoparticles, Dufour number and Soret number?

## Mathematical formulation

When energy flux due to concentration gradient and mass flux due to temperature gradient are significant, the dynamics of water conveying copper nanoparticles over a horizontal surface subject to magnetic field of strength $B_o$ inclined at an angle $\gamma$ was formulated. Due to the stretching at the wall, stretching velocity is assumed to be $u_w = \lambda U_ox$ where $\lambda <0$ implies shrinking while $\lambda >0$ implies stretching at the wall $y = 0$. The chemical reaction that occurs in the transport phenomenon was model as first order where the rate of the chemical reaction is $Kc^*$. Following Bachok et al.^39^, the governing equation suitable to investigate the aforementioned transport phenomenon is

1 $$\begin{aligned} u_x + v_y= & {} 0, \end{aligned}$$

2 $$\begin{aligned} uu_x + vu_y= & {} \frac{\mu _{nf}}{\rho _{nf}}u_{yy} - \frac{\sigma _{nf}B_o^2u}{\rho _{nf}}sin^2(\gamma ), \end{aligned}$$

3 $$\begin{aligned} uT_x + vT_y= & {} \frac{\kappa _{nf}}{(\rho c_p)_{nf}} T_{yy} + \frac{\sigma _{nf}B_o^2}{(\rho c_p)_{nf}}u^2sin^2(\gamma ) + \frac{Q_e^*(T_f - T_\infty )}{(\rho c_p)_{nf}}exp\left( -ny\sqrt{\frac{a}{\vartheta _f}}\right) + \frac{D_mk_t}{c_sc_p}C_{yy}, \end{aligned}$$

4 $$\begin{aligned} uC_x + vC_y= & {} D_BC_{yy} - K_c^*(C - C_\infty ) + \frac{D_mk_t}{t_m}T_{yy}. \end{aligned}$$

Suitable boundary conditions are

5 $$\begin{aligned}&u = \lambda u_w(x), \;\;\ v = v_w, \;\;\ T = T_w, \;\;\ C = C_w \;\;\ at \;\;\ y=0 \end{aligned}$$

6 $$\begin{aligned}&u \rightarrow 0, \;\;\;\;\;\;\; T \rightarrow T_\infty , \;\;\;\;\;\;\; C \rightarrow C_\infty \;\;\ as \;\;\ y \rightarrow \infty \end{aligned}$$

The model proposed by Graham^31^ and Gosukonda et al.^32^ for the ratio of dynamic viscosity of the nanofluid to the dynamic viscosity of base fluid defined as

7 $$\begin{aligned} \frac{\mu _{nf}}{\mu _{bf}} = 1 + 2.5\phi + 4.5\left[ \frac{1}{\frac{h}{d_p}\left( 2 +\frac{h}{d_p} \right) \left( 1 +\frac{h}{d_p} \right) ^2}\right] , \end{aligned}$$

where the radius of nanoparticle is $d_p$ and the inter-particle spacing is *h* was adopted. The effective nanofluid properties are given by

$$\begin{aligned}(\rho c_p)_{nf} = (1 - \phi )(\rho Cp)_{f} + \phi (\rho Cp)_{s}, \;\;\; \rho _{nf} = (1 - \phi )\rho _{f} + \phi \rho _{s},\end{aligned}$$

8 $$\begin{aligned} \frac{\sigma _{nf}}{\sigma _{f}} = \left[ 1 + \frac{3\left( \frac{\sigma _s}{\sigma _f} - 1 \right) \phi }{\left( \frac{\sigma _s}{\sigma _f} + 2 \right) - \left( \frac{\sigma _s}{\sigma _f} - 1 \right) \phi } \right] , \;\;\; \frac{\kappa _{nf}}{\kappa _{f}} = \frac{\kappa _{s} + 2\kappa _{f} - 2\phi (\kappa _{f} - \kappa _{s})}{\kappa _{s} + 2\kappa _{f} + \phi (\kappa _{f} - \kappa _{s})}, \end{aligned}$$

where $\phi$ is the solid volume fraction, $\mu _f$ is the dynamic viscosity of the base fluid, $\rho _f$ and $\rho _s$ are the densities, $(\rho c_p)_f$ and $(\rho c_p)_s$ are the heat capacitance, $\kappa _f$ and $\kappa _s$ are the thermal conductivities and $\sigma _f$ and $\sigma _s$ are the electrically conductivities of the base fluid and nanoparticles respectively. Prior to one of the conclusions by Buongiorno et al.^33^ on the thermal conductivity of nanofluids, the model proposed by Maxwell^34^ was adopted to incorporate the enhancement in the thermal conductivity of *Cu*-Water nanofluid. Next is to introduce the following variables

$$\begin{aligned}\eta = y\sqrt{\frac{a}{\vartheta _f}}, \;\ u(x,y) = ax\frac{df}{d\eta }, \;\ v(x,y) = -(a\vartheta _f)^{1/2}f(\eta ), \;\ \theta (\eta ) = \frac{T - T_\infty }{T_w - T_\infty }, \;\ \phi (\eta ) = \frac{C - C_\infty }{C_w - C_\infty }, \end{aligned}$$

where $D_f$ is the Dufour number, $S_r$ is the Soret number, Prandtl number $P_r$, magnetic field parameter *M*, and porosity parameter *P*, heat source/sink parameter $Q_e$, Lewis number $L_e$, viscous dissipation term $E_c$, and suction *S* are defined as

$$\begin{aligned} P_r= & {} \frac{\mu _{f} Cp_{f}}{\kappa _{f}}, \;\;\; A_1 = 1 + 2.5\phi + 4.5\left[ \frac{1}{\frac{h}{d_p}\left( 2 +\frac{h}{d_p} \right) \left( 1 +\frac{h}{d_p} \right) ^2}\right] , \;\;\ A_2 = 1 - \phi + \phi \frac{\rho _{s}}{\rho _{f}}, \\ A_3= & {} \left[ 1 + \frac{3\left( \frac{\sigma _s}{\sigma _f} - 1 \right) \phi }{\left( \frac{\sigma _s}{\sigma _f} + 2 \right) - \left( \frac{\sigma _s}{\sigma _f} - 1 \right) \phi } \right] , \;\;\ M = \frac{B_o^2\sigma _{f}}{a \rho _{f}}, \;\; Q_e = \frac{Q_e^*}{a(\rho Cp)_{f}}, \;\;\; L_e = \frac{\alpha }{D_m}, \\ A_5= & {} 1 - \phi + \phi \frac{(\rho Cp)_{s}}{(\rho Cp)_{f}}, \;\;\; A_4 = \frac{\kappa _{s} + 2\kappa _{f} - 2\phi (\kappa _{f} - \kappa _{s})}{\kappa _{s} + 2\kappa _{f} + \phi (\kappa _{f} - \kappa _{s})}, \;\;\ E_c = \frac{u_w^2}{Cp_f(T_w - T_\infty )}, \end{aligned}$$

9 $$\begin{aligned} D_f = \frac{D_mk_t}{c_sc_p \vartheta }\frac{(C_w - C_\infty )}{(T_w - T_\infty )},\;\;\ S_r = \frac{D_mk_t}{t_m \alpha }\frac{(T_w - T_\infty )}{(C_w - C_\infty )}, \;\;\ K_c = \frac{K_c^*}{a}, \;\;\ S = \frac{v_w}{-(a\vartheta _f)^{1/2}}. \end{aligned}$$

The dimensionless governing equation is now of the form

10 $$\begin{aligned} \frac{A_1}{A_2}\frac{d^3f}{d\eta ^3} - \frac{df}{d\eta }\frac{df}{d\eta } + f\frac{d^2f}{d\eta ^2} - \frac{A_3}{A_2}M\frac{df}{d\eta }Sin^2(\gamma )= & {} 0, \end{aligned}$$

11 $$\begin{aligned} \frac{A_4}{A_5}\frac{d^2\theta }{d\eta ^2} + P_rf\frac{d\theta }{d\eta } + P_r\frac{A_3}{A_5}ME_c\frac{df}{d\eta }\frac{df}{d\eta }sin^2(\gamma ) + \frac{Q_e}{A_5}exp(-n\eta ) + D_f\frac{d^2\phi }{d\eta ^2}= & {} 0, \end{aligned}$$

12 $$\begin{aligned} \frac{d^2\phi }{d\eta ^2} + P_rL_ef\frac{d\phi }{d\eta } - P_rL_eK_c\phi + L_eS_r\frac{d^2\theta }{d\eta ^2}= & {} 0. \end{aligned}$$

Subject to

13 $$\begin{aligned}&f = S, \;\;\;\;\ \frac{df}{d\eta } = \lambda , \;\;\;\;\ \theta = 1, \;\;\;\;\ \phi = 1 \;\;\ at \;\;\ \eta = 0 \end{aligned}$$

14 $$\begin{aligned}&\frac{df}{d\eta } \rightarrow 0, \;\;\;\;\ \theta \rightarrow 0, \;\;\;\;\ \phi \rightarrow 0 \;\;\; as \;\;\; \eta \rightarrow \infty . \end{aligned}$$

Skin friction coefficient $Cf_x$, Nusselt number $Nu_x$, and Sherwood number $Sh_x$ are defined as

$$\begin{aligned}Cf_x = \frac{\tau _w}{\rho _f u_w^2} = \frac{\mu _{nf}}{\rho _f a^2x^2}\frac{\partial u}{\partial y}, \;\;\;\ Nu_x = \frac{xq_w}{\kappa _f(T_w - T_\infty )} = \frac{-x\kappa _{nf}}{\kappa _f(T_w - T_\infty )}\frac{\partial T}{\partial y}, \end{aligned}$$

15 $$\begin{aligned} Sh_x = \frac{xq_m}{D_B(C_w - C_\infty )} = \frac{-xD_B}{D_B(C_w - C_\infty )}\frac{\partial C}{\partial y}, \end{aligned}$$

where Reynold number $\sqrt{Re_x} = \frac{a^{1/2}x}{\vartheta _f^{1/2}}$, shear stress $\tau _w$, heat flux $q_w$, mass flux $q_m$, $\rho _f = 997.1$, $(Cp)_f = 4179$, $\kappa _f = 0.613$, $\sigma _f = 5.5\times 10^{-6}$, $\rho _s = 8933$, $(Cp)_s = 385$, $\kappa _s = 401$, and $\sigma _s = 5.96\times 10^{7}$. These values are extracted from Wakif et al.^35^, Saidi & Karimi^36^, Khoshvaght-Aliabadi & Hormozi^37^, Wan et al.^38^, and Bachok *et al.*^39^. The dimensionless local skin friction, heat transfer rate, and mass transfer rate are

16 $$\begin{aligned} Cf_x\sqrt{Re_x} = f''(0), \;\;\;\;\; \frac{Nu_x}{\sqrt{Re_x}} = - \theta '(0), \;\;\;\;\; \frac{Sh_x}{\sqrt{Re_x}} = - \phi '(0). \end{aligned}$$

**Table 1:** Comparison of $-\theta '(0)$ for different values of $P_r$ when $\frac{A_1}{A_2} = \frac{A_4}{A_5} = 1$, $M = 0$, $Q_e =S = 0$, $\lambda = 1$, and $L_e = D_f = 0$.

$P_r$	Hamad^44^	Wang^45^	Gorla and Sidawi^46^	Khan and Pop^47^	Present study $\eta _\infty = 100$
0.07	0.06556	0.06557102	0.0656	0.0663	0.065622583128431
0.2	0.16909	0.16908943	0.1691	0.1691	0.169088618141040
0.7	0.45391	0.45390994	0.5349	0.4539	0.453916816202306
2	0.91136	0.91136321	0.9114	0.9113	0.911361392450143
7	1.89540	1.89541263	1.8905	1.8954	1.895420199527724
20	3.35390	3.35380245	3.3539	3.3539	3.353934997970873
70	6.46220	6.46219257	6.4622	6.4621	6.462312632472948

## Method of solution, results and discussion

**Figure 1:**
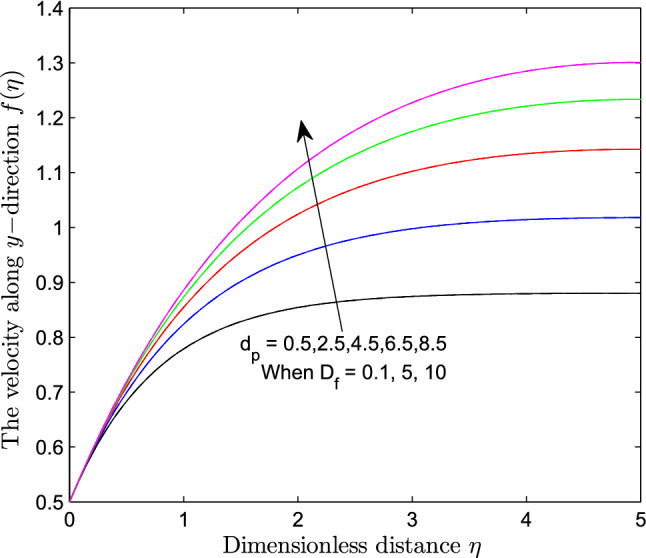
Variation in the species (concentration) within the domain when $D_f = 0.1$.

**Figure 2:**
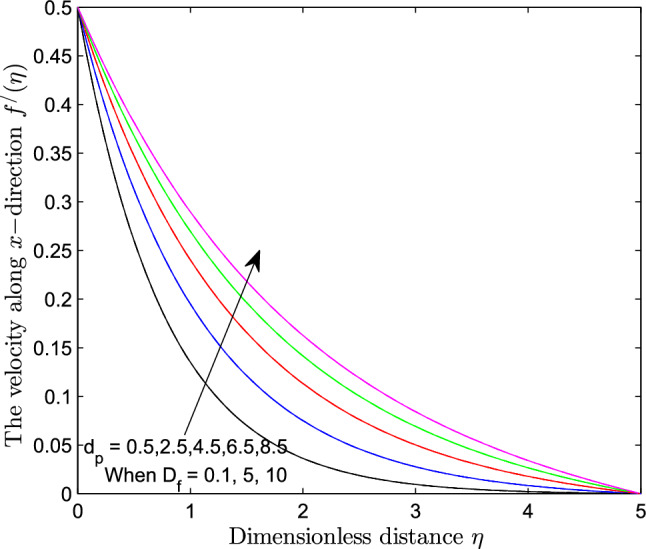
Variation in the species (concentration) within the domain when $D_f = 10$.

The method of superposition (Na^40^) was used to obtain the system of first order IVP for Eqs. (10)–(14). The numerical solution of the corresponding IVP was obtained using MATLAB based bvp4c package suggested by Gokhan^41^, Kierzenka and Shampine^42^, and Ali Umit Keskin^43^. Reliability and validity of solution was established by comparing the limiting case of this present study with that of Hamad^44^ at various values of Prandtl number. As shown in Table 1, good agreement is seen, hence further analysis is reliable. The simulation was carried out for fixed magnetic field strength $M = 0.5$, inclination of the magnetic field $\gamma = 30^o$, inter-particle spacing $h = 1$, Lewis number $L_e = 0.2$, Prandtl number $P_r = 6.2$, chemical reaction parameter $K_c = 0.5$, heat source parameter $Q_e = 0.5$, intensity of heat source $n = 0.5$, viscous dissipation term—Eckert number $E_c = 0.3$, stretching related parameter $\lambda = 0.5$, and suction $S = 0.5$. The answer to the remaining research questions was obatined when $M = 0.5$, $\gamma = 30^o$, $h=1$, $L_e = 0.2$, $P_r = 6.2$, $K_c = 0.5$, $Q_e = 0.5$, $n = 0.5$, $E_c = 0.3$, $\phi = 0.02$, $S = 0.5$, and $\lambda = 0.5$. Using the technique called linear regression through the data point suggested by Shah et al.^50^, Wakif et al.^51^, and Animasaun et al.^52^, the analysis presented as Tables 2, 3, 4 and 5 reveal that a larger radius of copper nanoparticles leads to negligible higher local skin friction coefficients. Firstly, when $S_r = 0.1$ and $D_f = 0.1$, as $d_p$ increases, local skin friction coefficients increases at the rate of 0.046678714, higher heat transfer rate of 0.019499889, and higher mass transfer rate of 0.004874409. In order to capture the significance of energy flux due to concentration gradient, the same analysis was carried out for $D_f = 10$; see Table 3. Percentage increase in $\frac{Nu_x}{\sqrt{Re_x}}$ with $d_p$ as $D_f$ changes from 0.1 to 10 was estimated $344.286288\%$. Comparative analysis of the data presented as Tables 4 and 5 and figures illustrated as Figs. 1 and 2 confirm that there exists a relationship between energy flux due to concentration gradient and mass flux due to temperature gradient.

**Table 2:** Variation in $Cf_x\sqrt{Re_x}$, $\frac{Nu_x}{\sqrt{Re_x}}$, and $\frac{Sh_x}{\sqrt{Re_x}}$ with $d_p$ when $\eta _\infty = 100$, $S_r = 0.1$, and $D_f = 0.1$.

$d_p$	$Cf_x\sqrt{Re_x}$	$\frac{Nu_x}{\sqrt{Re_x}}$	$\frac{Sh_x}{\sqrt{Re_x}}$
0.5	$-0.653806450541409$	2.598649354329436	1.253971131437740
2.5	$-0.469235981875199$	2.658463112787918	1.271088881799449
4.5	$-0.361939515077478$	2.691156656593007	1.283009832479554
6.5	$-0.305655478525104$	2.744891132958165	1.289010352971316
8.5	$-0.268809557314600$	2.750434236178156	1.293754488967823
$S_{lp}$	0.046678714	0.019499889	0.004874409

**Table 3:** Variation in $Cf_x\sqrt{Re_x}$, $\frac{Nu_x}{\sqrt{Re_x}}$, and $\frac{Sh_x}{\sqrt{Re_x}}$ with $d_p$ when $\eta _\infty = 100$, $S_r = 0.1$ and $D_f = 10$.

$d_p$	$Cf_x\sqrt{Re_x}$	$\frac{Nu_x}{\sqrt{Re_x}}$	$\frac{Sh_x}{\sqrt{Re_x}}$
0.5	$-0.653806450627237$	$-8.856154914369313$	1.471944690641082
2.5	$-0.469235982235743$	$-8.531505838195230$	1.483819380613837
4.5	$-0.361939515178205$	$-8.330285252873948$	1.492449292624451
6.5	$-0.301600963981252$	$-8.217430056390326$	1.498031241838610
8.5	$-0.262740377859659$	$-8.146839475383260$	1.501956963392201
$S_{lp}$	0.047488358	0.086635333	0.00371182

**Table 4:** Variation in $Cf_x\sqrt{Re_x}$, $\frac{Nu_x}{\sqrt{Re_x}}$, and $\frac{Sh_x}{\sqrt{Re_x}}$ with $d_p$ when $\eta _\infty = 100$, $S_r = 10$ and $D_f = 0.1$.

$d_p$	$Cf_x\sqrt{Re_x}$	$\frac{Nu_x}{\sqrt{Re_x}}$	$\frac{Sh_x}{\sqrt{Re_x}}$
0.5	$-0.653806450627237$	3.052651035544433	$-3.874684483749367$
2.5	$-0.469235982235743$	3.120598790031860	$-3.954088416453056$
4.5	$-0.361939515178206$	3.157453355416220	$-3.988894824862508$
6.5	$-0.301600963981252$	3.174211445520884	$-3.998815684760363$
8.5	$-0.262740377859659$	3.181969713124626	$-3.998798514798066$
$S_{lp}$	0.047488358	0.015612501	$-0.014647767$

**Table 5:** Variation in $Cf_x\sqrt{Re_x}$, $\frac{Nu_x}{\sqrt{Re_x}}$, and $\frac{Sh_x}{\sqrt{Re_x}}$ with $d_p$ when $\eta _\infty = 100$, $S_r = 10$ and $D_f = 10$.

$d_p$	$Cf_x\sqrt{Re_x}$	$\frac{Nu_x}{\sqrt{Re_x}}$	$\frac{Sh_x}{\sqrt{Re_x}}$
0.5	$-0.653806450603498$	0.792715106318922	0.495439010086529
2.5	$-0.469235982226111$	0.792495006570055	0.508203934009412
4.5	$-0.361939515173113$	0.794097753963524	0.517949200635406
6.5	$-0.301600963978340$	0.795989676393173	0.524492344750289
8.5	$-0.262740377857890$	0.797735022943715	0.529210531128244
$S_{lp}$	0.047488358	0.000676725	0.004191573

**Figure 3:**
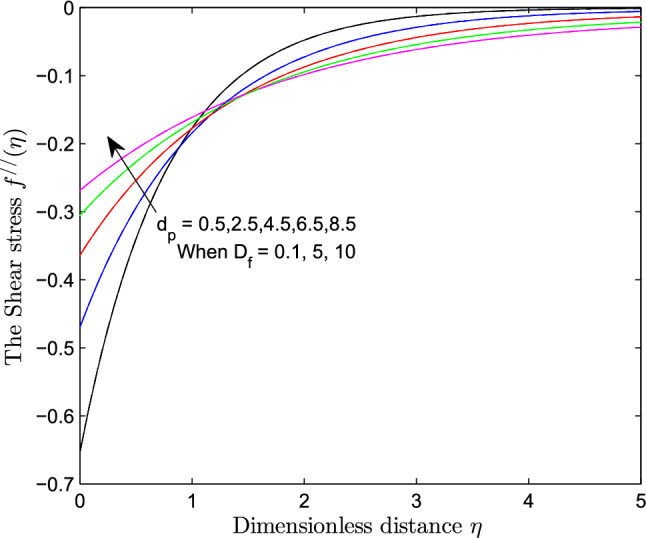
Variation in the velocity along $y-$direction with $d_p$ at various values of $d_f$.

**Figure 4:**
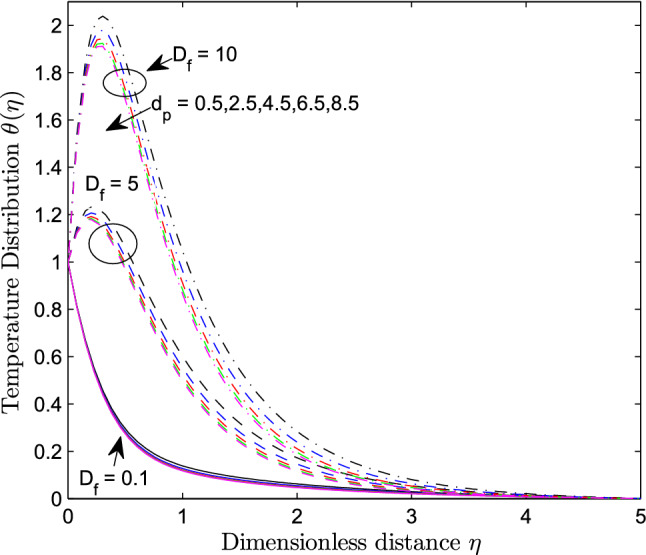
Variation in the velocity along $x-$direction with $d_p$ at various values of $d_f$.

**Figure 5:**
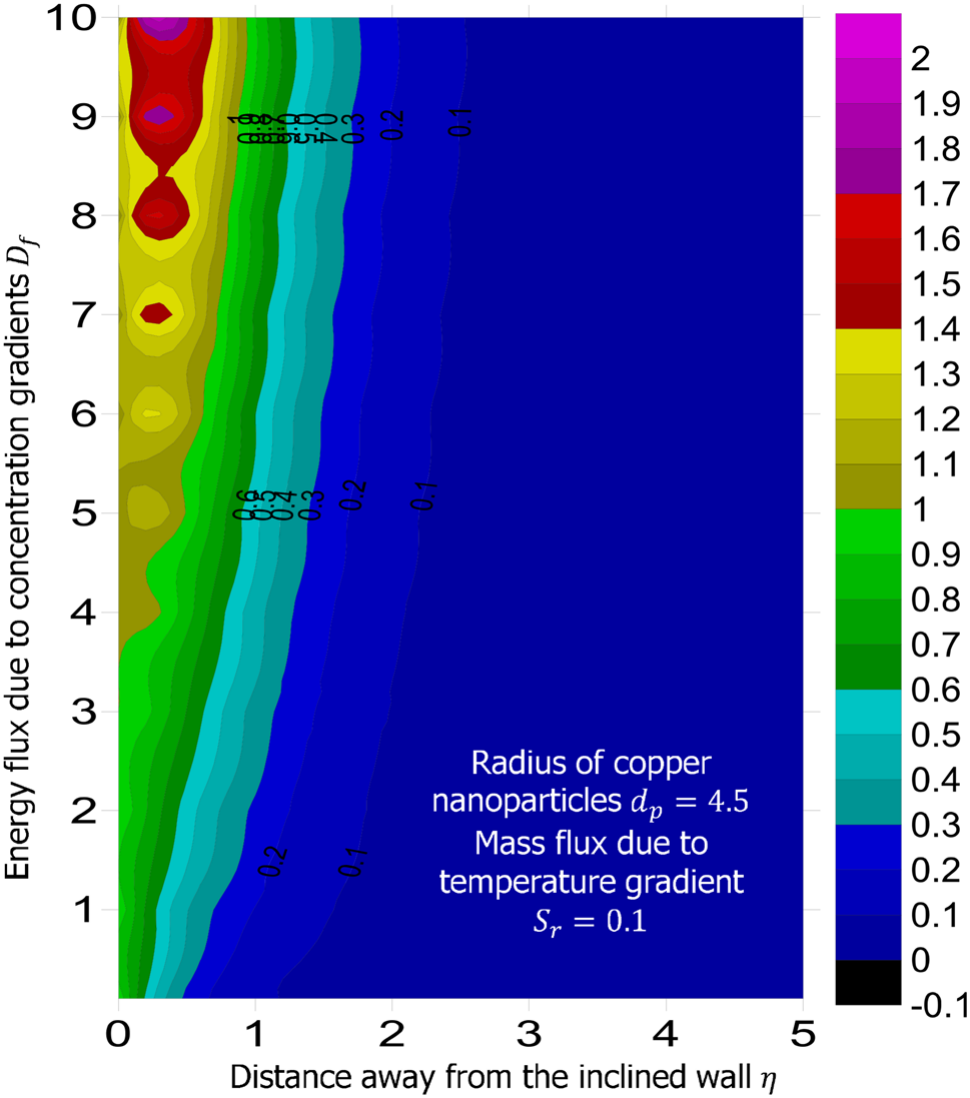
Variation in shear stress within the domain due to $d_p$ at various values of $d_f$.

**Figure 6:**
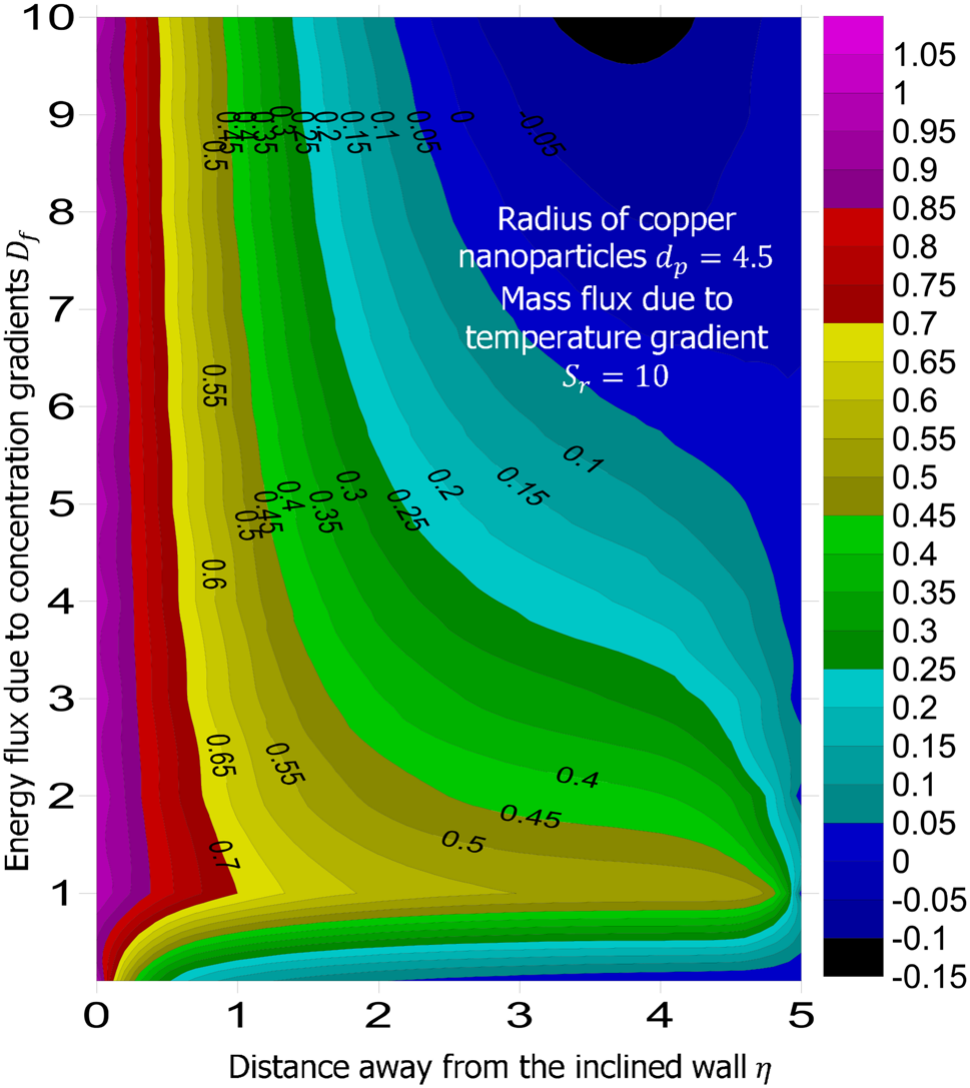
Variation in the distribution of temperature within the domain due to $d_p$ at various values of $d_f$.

**Figure 7:**
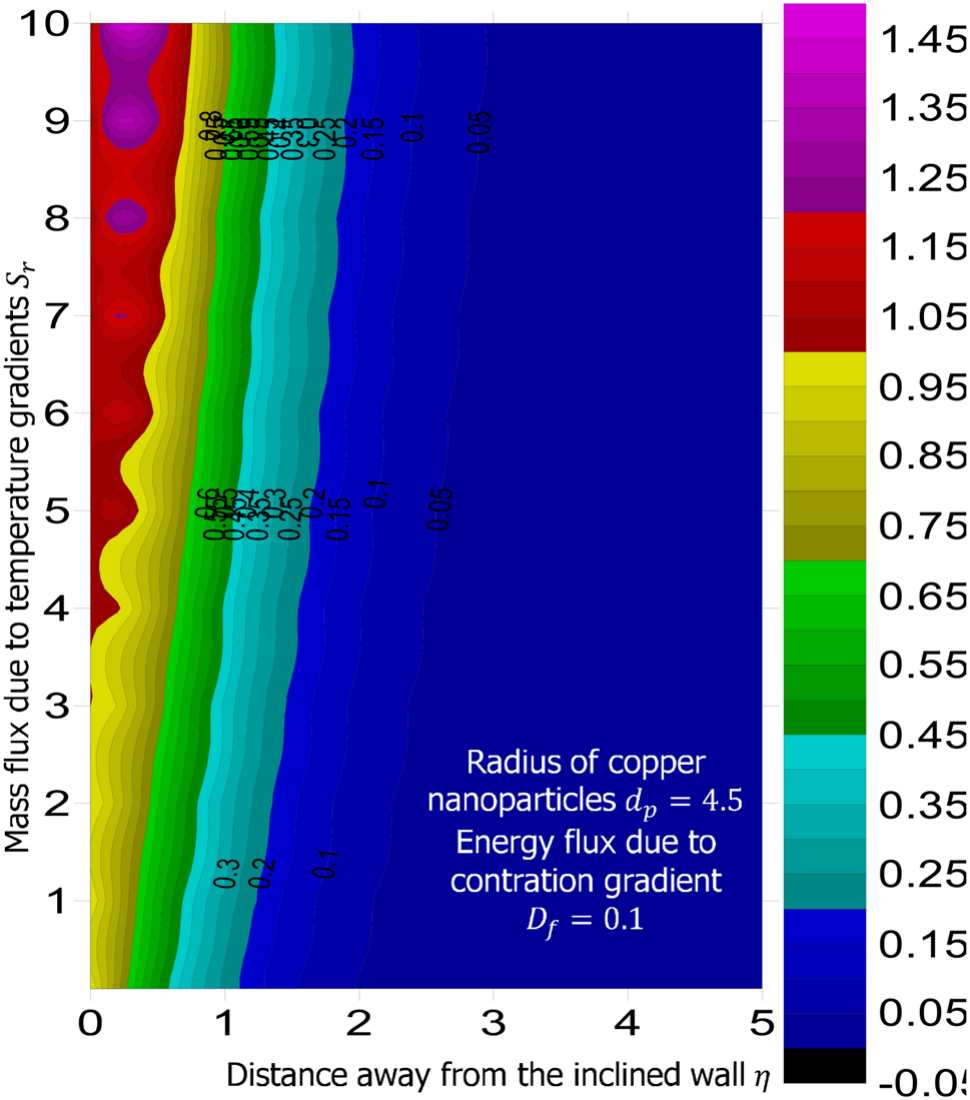
Variation in the distribution of temperature within the domain when $S_r = 0.1$.

**Figure 8:**
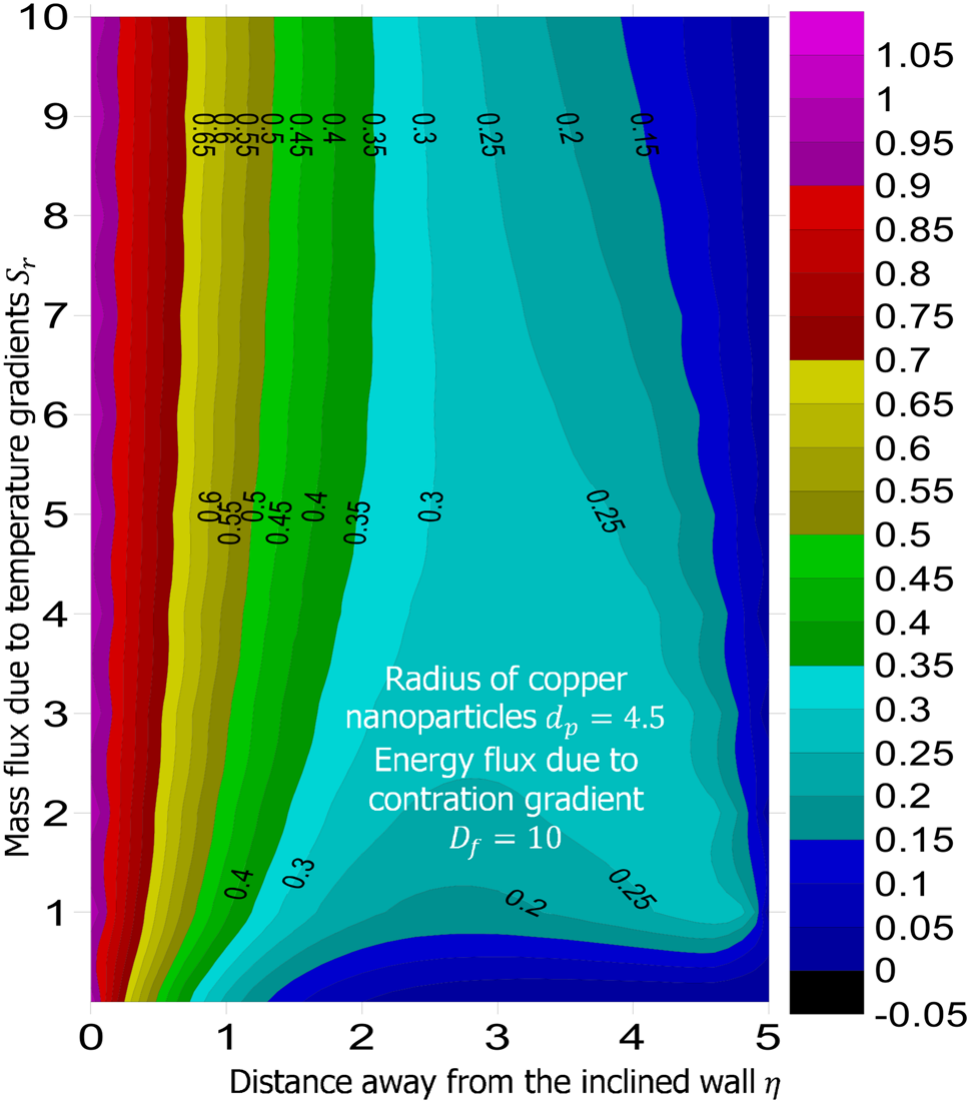
Variation in the distribution of temperature within the domain when $S_r = 10$.

When $S_r = 0.1$, the significance of increasing radius of copper nanoparticles $d_p$ and energy flux due to concentration gradient $D_f$ were examined. The outcome of the analysis presented as Figs. 3 and 4 show that the velocity of the transport phenomenon increases with a larger radius of nanoparticles. Meanwhile, an increment in the friction across the layers near the inclined surface is observed through an increase in the radius of nanoparticles as shown in Fig. 5. This can be associated with the fact that increasing the diameter of nanoparticles in water as pointed out by Namburu et al.^24^ corresponds to a decrease in the nanofluid’s viscosity. Not only that, the outcome of an examination of (i) ethylene glycol and alumina nanoparticles mixture (ii) water and CuO nanoparticles mixture by Pastoriza-Gallego et al.^48,49^ shows that higher viscosity is bound to occur as particle size diminishes. It is worth deducing from Figs. 3, 4, and 5 that the velocity is the same at all the levels of heat energy flux due to concentration gradient $D_f$. However, in the case of distribution of temperature illustrated as Fig. 6, decreases negligibly with $d_p$ when $D_f$ is small in magnitude. When $D_f = 5$ and $D_f = 10$, the observed decrease in the temperature distribution due to the higher radius of nanoparticles is ascertained. Further exploration of temperature distribution for $0.1 \le D_f \le 10$ was achieved through kriging gridding method package in Surfer plot version 11.1.719 as shown in Figs. 7 and 8. When the transfer of species due to temperature gradient is small in magnitude optimal temperature is seen a few distances away from the inclined surface at larger values of $D_f$. Now, when mass flux due to temperature gradient is sufficiently large, optimal temperature, although it is small in magnitude is seen at all the levels of energy flux due to the concentration gradient.

## Conclusion

Attempt had been made to examine the significance of increasing radius of nanoparticles, energy flux due to concentration gradient, and mass flux due to temperature gradient in the dynamics of chemically reactive fluid subject to suction and inclined magnetic strength. Based on the analysis, it is worth concluding that

1. at all the levels of energy flux due to concentration gradient, reduction in the viscosity of water-based nanofluid due to a higher radius of copper nanoparticles causes an enhancement of the velocity.
2. significance decrease in distribution of temperature across the domain due to increasing radius of copper nanoparticles is achievable when energy flux due to concentration gradient is sufficiently large in magnitude.
3. when mass flux due to temperature gradient is highly negligible, optimal temperature is also observable when energy flux due to concentration gradient is sufficiently large. Reverse is the case when mass flux due to temperature gradient is sufficiently large as optimal temperature is ascertained at all levels of energy flux due to concentration gradient.
4. reduction in the mass transfer rate $\frac{Sh_x}{\sqrt{Re_x}}$ due to higher radius of copper nanoparticles is guaranteed when the transfer of species (mass) due to temperature gradient is sufficiently large but transfer of heat energy due to concentration gradient is highly negligible.
5. the emergence of both energy flux and mass flux due to gradients in concentration and temperature affect the distribution of temperature and concentration at the free stream.
